# 

**Published:** 1881-05-20

**Authors:** 


### EDITORIALS AND MISCELLANEOUS.

☞ **Subscribers are earnestly requested to remit their dues.**

### *EDITORIAL NOTICES.*

THE Medical Society of North Carolina meets at Ashville on Tuesday, May 31st.

DR. J. B. UNDERWOOD, an old and respected physician, died recently at his home in Cave Springs, Ga.

DR. CHAS. MAGILL, an eminent physician of Richmond, Virginia, died May 5th, 1881, aged 75 years.

*Plague.*—Reports from Turkey state that the plague is prevailing with awful and increasing severity at Bagdad.

*Dr. Ray Dead.*—Dr. Isaac Ray, of Philadelphia, a distinguished writer and physician, died March 31st, 1881, aged 75 years.

*Illustrated Scientific News.*—We are well pleased with the Illustrated Scientific News, a monthly published by Munn & Co., New York. Terms, $1.50 per annum.

*Death from Vaccination.*—Twelve deaths are reported in New York during last summer from vaccination—from erysipelas in most cases, resulting from impure matter.

DR. COWLING, Editor of Louisville Medical News and Professor of Surgery in Louisville Medical College, died of a rheumatic affection, on April 2d, 1881, aged 42 years.

*Tennessee State Medical Society.*—The late meeting of the Tennessee State Medical Society was very poorly attended, caused, we are told, by division or dissension in that body.

*Doctors Take Notice* that the property advertised by J. A. Ansley, in this Journal, is a bargain and a rare chance to obtain a cheap home in a city which is rapidly growing, and where, in a few years, such property will be double its present price.

*Southern Medical College.*—The prospects of this new institution are very flattering. The arrangements for the hospital in connection with the Institute are progressing favorably, and other facilities are being provided for imparting medical information second to no school in the country. Southern students need not now go North to obtain a medical education, and Northern students, especially those who have reason to prefer a mild climate in which to pursue their studies, will do well to take lectures at the Southern Medical College.

THE Association of Medical Editors recently held in Richmond, elected the following gentlemen as officers for the ensuing year: L. B. Edwards, M. D., of Virginia, President; Ralph Wallace, M. D., of D. C, Vice-President, and Dr. D. S. Reynolds, of Kentucky, Secretary.

OUR thanks returned for a sample of *Rogers' Bird's Eye-Views*, containing, on a sheet not larger than an ordinary newspaper—Rules for spelling and punctuating; using capitals; letter writing; spelling of 25,000 words, 20,000 synonyms, etc. A very useful and valuable sheet to everybody. Send 25 cents to L. H. Rogers, publisher, New York city.

*Tannerism.*—A lady in Iowa City—Miss Hattie Duell, voluntarily abstained from food for forty-seven days and died from inanition on the 10th inst. It was a deliberate and most remarkable suicide. Her symptoms very much resembled those experienced by Dr. Tanner. In the latter case it was supposed that the will-power of the man greatly contributed to the maintenance of life, but in the case of Miss Duell there was a desire for death; by which it is shown that the natural powers of life will suffice to keep up the body for forty-seven days at least.

RECEIPTED, 1880.—J. F. Stoddard, A. C. Coleman, W. F. Gresham, R. D. Lucas. 1881.—L. N. Hyten, J. W. Baker, R. H. Davis, S. M. Hogan, M. E. Demaret, J. H. Rentz, T. J. Jones, J. D. Moore, H. Perdue, S. B. Ragon, J. F. Davis, W. T. Mathews, T. H. Roberds, J. T. McDowell, C. C. Jones, R. D. Jackson.

